# Draft genome sequence of *Leptospira* sp. SA-E8, isolated from a farmland soil

**DOI:** 10.1128/mra.00929-25

**Published:** 2025-10-31

**Authors:** Amin Sonam, Asif Hameed, Paul Stothard, Jason R. Grant, Ananthapadmanabha Bhagwath Arun

**Affiliations:** 1 Division of Microbiology and Biotechnology, Yenepoya Research Centre, Yenepoya (Deemed to be University) 231500, Mangalore, India; 2 Department of Agricultural, Food and Nutritional Science, University of Alberta 3158, Edmonton, Alberta, Canada; 3 Yenepoya Institute of Arts, Science, Commerce and Management 552369 , Mangalore, India; University of Strathclyde, Glasgow, United Kingdom

**Keywords:** *Leptospira*, genomic analysis, genome sequence, spirochaete, virulence

## Abstract

We report the draft genome sequence of *Leptospira* sp. SA-E8, isolated from a farmland soil in India. SA-E8 shared 88.8% OrthoANI and 36.2% dDDH scores with *L. andrefontaineae* PZF11-2^T^, indicating distinct taxonomic placement of the isolate. *In silico* analysis predicted potential drug resistance and hemolytic traits in SA-E8.

## ANNOUNCEMENT

*Leptospira* sp. SA-E8, belonging to the phylum *Spirochaetota,* was isolated from a farmland soil of Karnataka, India (14°17′41.7ʺN 74°50′16.2ʺE). The topsoil (50 g) was collected in a sterile container, mixed with 100 mL of Ellinghausen–McCullough–Johnson–Harris (EMJH) basal medium, and left to settle at 25°C–27°C for 30 min. The supernatant was centrifuged (250 × *g*, 10 min, 25°C), filtered through 0. 22-µm filter, and inoculated (0.5 mL) into 2 mL of enriched EMJH liquid medium containing 200 µg/mL 5′ fluorouracil (1). The static culture was incubated under darkness and aerobic conditions at 30°C for 2 months. The cells were harvested by centrifugation (10,000 rpm, 5 min, 25°C), and DNA was extracted using the QIAamp DNA mini kit (Qiagen). The quality and quantity of DNA were assessed using nanodrop spectrophotometry and gel electrophoresis. The genome was sequenced using the Illumina Nextseq2000 with the read chemistry of 2 × 150 bp. DNA was enzymatically fragmented followed by library preparation using QIAseq FX DNA Library Kit (Qiagen). The QC for raw Illumina reads was assessed using FastQC (2). Adaptor and low-quality reads were removed using the fastp tool (v. 0.23.2) (3). The genome was assembled using SPAdes (v. 3.15.3) (4), and the quality of assembly was analyzed with *Leptospira* CheckM (v. 1.2.4) marker set (5). The genome was annotated using Prokaryotic Genome Annotation Pipeline (PGAP v. 6.10) of NCBI (6) The intergenomic distance calculation and digital DNA-DNA hybridization were done through Type (Strain) Genome Server (TYGS v. 401) (7). Orthologous average nucleotide identity was calculated using the OrthoANI (v. 0.93.1) (8). The genes predicted to be responsible for intrinsic resistance were identified through PATRIC Bacterial and Viral Bioinformatics Resource Center (BV-BRC) (9). Unless otherwise noted, default parameters were used for all software.

The number of raw read pairs obtained was 1,535,731 and the total raw data generated was 457.7 M bp with the read length of 2 × 150 bp. CheckM completeness and contamination were 81.71% and 12.1%, respectively. As per PGAP analysis, the draft genome (4,406,931 bp) of SA-E8 contained 43% G + C content, 656 contigs (contig N50: 597.4 kb), 4,532 genes, 4,470 protein CDS, and 47 genes coding for RNA. SA-E8 shared the highest OrthoANI (88.8%) and dDDH (36.2%) values with *L. andrefontaineae* PZF11-2^T^ (RQEY00000000.1) (10, 11). The OrthoANI and dDDH values are well below the recommended cutoff to delineate the isolate at the species level (12). BV-BRC analysis predicted 25 drug-resistant genes. The genomic data indicate possible drug resistance and virulence attributes of SA-E8.

## Data Availability

The 16S rRNA gene and whole genome sequences of *Leptospira* sp. SA-E8 have been deposited at GenBank under the accession PX285705 and JBNURY000000000, respectively. The raw reads have been deposited under BioProject ID PRJNA1261035, SRA accession SRX29909408, and BioSample accession SAMN48410458.
